# Versatility of Hydrogels: From Synthetic Strategies, Classification, and Properties to Biomedical Applications

**DOI:** 10.3390/gels8030167

**Published:** 2022-03-07

**Authors:** Zubair Ahmad, Saad Salman, Shahid Ali Khan, Abdul Amin, Zia Ur Rahman, Youssef O. Al-Ghamdi, Kalsoom Akhtar, Esraa M. Bakhsh, Sher Bahadar Khan

**Affiliations:** 1 Department of Chemistry, University of Swabi, Swabi 23561, Pakistan; za3724364@gmail.com (Z.A.); abdulamin995@gmail.com (A.A.); ziaurrahman100@yahoo.com (Z.U.R.); 2 Faculty of Pharmacy, Capital University of Science and Technology, Islamabad 44000, Pakistan; saad.salman@pharm.uol.edu.pk; 3 Department of Chemistry, School of Natural Sciences, National University of Science and Technology (NUST), Islamabad 44000, Pakistan; 4 Department of Chemistry, College of Science Al-Zulfi, Majmaah University, Al-Majmaah 11952, Saudi Arabia; yo.alghamdi@mu.edu.sa; 5 Department of Chemistry, Faculty of Science, King Abdulaziz University, Jeddah 21589, Saudi Arabia; kaskhan@kau.edu.sa (K.A.); ibakhsh@kau.edu.sa (E.M.B.); 6 Center of Excellence for Advanced Materials Research, King Abdulaziz University, Jeddah 21589, Saudi Arabia

**Keywords:** hydrogels, synthetic strategies, classification, applications, wound healing, drug delivery

## Abstract

Hydrogels are three-dimensional, cross-linked, and supramolecular networks that can absorb significant volumes of water. Hydrogels are one of the most promising biomaterials in the biological and biomedical fields, thanks to their hydrophilic properties, biocompatibility, and wide therapeutic potential. Owing to their nontoxic nature and safe use, they are widely accepted for various biomedical applications such as wound dressing, controlled drug delivery, bone regeneration, tissue engineering, biosensors, and artificial contact lenses. Herein, this review comprises different synthetic strategies for hydrogels and their chemical/physical characteristics, and various analytical, optical, and spectroscopic tools for their characterization are discussed. A range of synthetic approaches is also covered for the synthesis and design of hydrogels. It will also cover biomedical applications such as bone regeneration, tissue engineering, and drug delivery. This review addressed the fundamental, general, and applied features of hydrogels in order to facilitate undergraduates, graduates, biomedical students, and researchers in a variety of domains.

## 1. Introduction

Three-dimensional (3D) hydrophilic polymer chains that expand in water and contain a high quantity of water are called hydrogels that were first reported by Wichterle and Lim in 1960 [1,2,3]. The hydrophilic functionalities are responsible for holding a large amount of water, and the network chains’ cross-linking allows them to retain water in their structure without being dissolved. Synthetic hydrogels have a higher capacity for water absorption than natural hydrogels. The amount of water in the hydrogel is determined by the polymer’s properties and the density of networking [4]. The inclusion of hydrophilic groups such as -NH_2_, -CONH, -SO_3_H, -CONH_2_, -COOH, and -OH contributes to the network’s hydrophilicity. For a substance to be a hydrogel, it must contain at least 10% water by weight or volume. Because of their high water content, hydrogels have a degree of elasticity that is close to natural tissue [5]. The degree of cross-linking, charge density, and type of monomer influences the sensitivity of hydrogels to external stimuli. The chemical and physical stimuli affect the nature of hydrogels; as a result, the hydrogels may store varying amounts of water. Solvent composition, temperature, electric and magnetic fields, pressure, and light intensity are examples of physical stimuli [6]. 

However, the hydrogels can revert to their initial configuration in most cases, such as conformational alterations are reversible, for example, a change in the chemical or biological stimuli such as pH, ions, [6], or specific compounds [7,8]. The magnitude of their response is proportional to the size of the external stimuli [9,10,11,12].

### 1.1. Classification of Hydrogel

Hydrogels are classified based on a variety of factors, including their origin or source, composition, structure configuration, cross-linking, network charge, durability, and response to external stimuli [13]. These sources of hydrogels are discussed below.

#### 1.1.1. Classification of Hydrogels Based on Source

##### Synthetic Hydrogels

**Figure 1:**
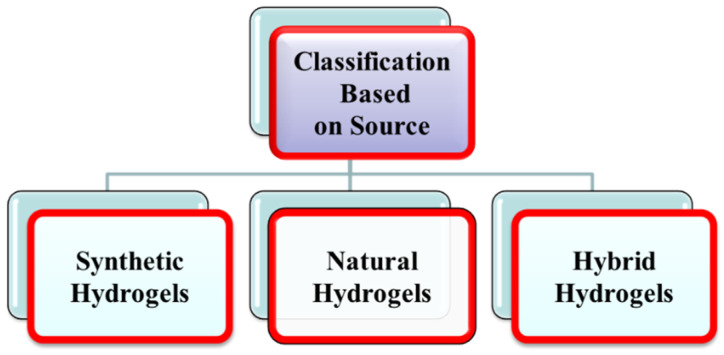
Classification of hydrogels based on source.

Polymeric synthetic hydrogels are three-dimensional swelling networks of covalently or ionically cross-linked hydrophilic homopolymers or copolymers hydrogels. Polymerization of various synthetic monomers yields synthetic hydrogels such as poly (hydroxyethyl methacrylate) PHEMA, polyethylene glycol (PEG) hydrogels, and polyacrylic acid (PAA) [14]. Figure 1 shows the many types of hydrogels dependent on the source.

##### Natural Hydrogels

Natural hydrogels with strong cell adhesion capabilities have traits such as biocompatibility and biodegradability. Protein and polysaccharides are two natural polymers used to make natural hydrogels. Proteins include collagen, lysozyme (LYZ), and gelatin, whereas polysaccharides include chitosan and alginate [15].

##### Hybrid Hydrogels

Hybrid hydrogels are made by combining natural and synthetic polymer hydrogels. Natural biopolymers such as collagen, chitosan, and dextran have been combined with synthetic polymers such as poly (N-isopropyl acrylamide) and polyvinyl alcohol. Hybrid hydrogels include CTN/PVA hydrogels and alginate/PEG hydrogels [16].

#### 1.1.2. Classification Based on Polymeric Composition

##### Homopolymeric Hydrogels

**Figure 2:**
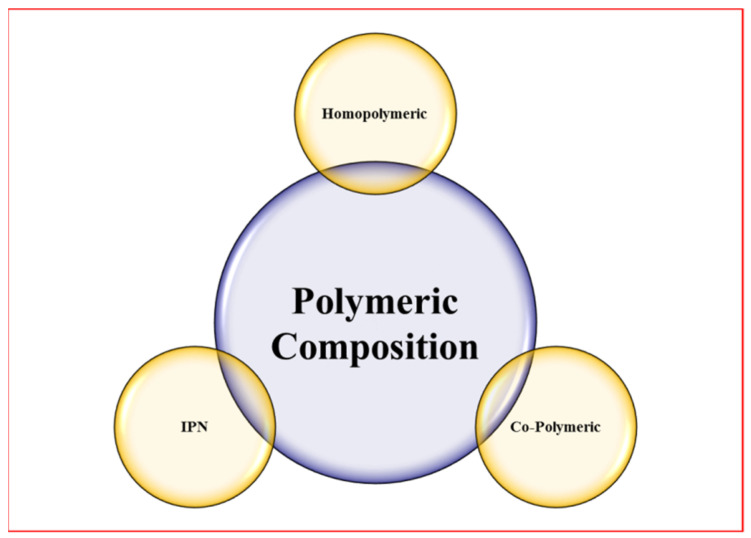
List of hydrogels classified based on polymeric composition. IPN stands for the interpenetrating network (two or more polymer cross-linked hydrogels).

Homopolymers are polymer networks made up of a single monomer species that have been cross-linked. The monomer of the same polymer network is the essential structural unit of homopolymers. The kind and nature of the monomer, as well as the processes of polymerization, determine whether a homopolymer is cross-linked or uncross-linked. For instance, poly(2-hydroxyethyl methacrylate) (PHEMA), poly(3-hydroxypropyl methacrylate) (PHPMA), and poly(hydroxyalkyl methacrylate) are examples of homopolymers. Drug carriers and contact lenses both utilize cross-linked homopolymers [17]. Poly(ethylene glycol) (PEG) and poly(*N*-vinyl-2-pyrrolidinone) (PNVP) are examples of uncross-linked homopolymeric hydrogels [18] (Figure 2).

##### Copolymeric Hydrogels

Covalently or ionically cross-linked copolymeric hydrogel networks are often non-water soluble. Two or more different monomers, at least one of which is hydrophilic, make up copolymeric hydrogels [19]. In the network chain, the hydrophilic groups of copolymer hydrogels can be grouped in a random, alternating, or block arrangement. Poly (HEMA-co-AA), poly (NVP-co-HEMA), and poly (NVP-co-HEMA) are some of the hydrogels available.

##### Multi-Polymer Integrating Polymer Network (IPN) Hydrogels

A hydrogel that consists of two independent and cross-linked chains of polymers, which may be natural or manufactured, and is arranged in a network shape is known as an interpenetrating polymer network (IPN). A semi-IPN is an IPN in which one component is a cross-linked polymer and the other is a non-crosslinked polymer [20]. Numerous researchers have reported a variety of IPNs and semi-IPNs derived from polysaccharides such as chitosan and its derivatives, poly (*N*-isopropyl acrylamide) (PNIPAM), PNVP, PVA, poly (ethylene oxide) (PEO), PEG, and poly (methacrylic acid) (PMAA). The IPN hydrogels are reported to have potential bio-applications [21].

#### 1.1.3. Classification Based on Cross-Linking

Hydrogels are categorized into chemical and physical cross-linking based on cross-linking. Because chemical cross-linked hydrogels are created through a chemical reaction, they have permanent links. Chemically cross-linked hydrogels include alginate-based hydrogels [22]. Polymer chain entanglements, hydrophobic interactions, and physical interactions such as hydrogen and ionic bonding can all result in physically cross-linked hydrogels. In contrast to chemically cross-linked hydrogels, which have permanent junctions, physically cross-linked hydrogels have temporary connections [15].

#### 1.1.4. Classification Based on Network Electrical Charge

##### Neutral Hydrogel/Non-Ionic Hydrogels

Non-ionic hydrogels, commonly referred to as neutral hydrogels, can have homopolymeric or copolymeric networks. In the creation of such hydrogels, there are no charged groupings. Various polymerization processes or manufacturing in the polymer chain can be used to make these hydrogels. A change in the system’s temperature can cause the neutral hydrogels to swell or shrink. In their polymer organizations, they feature ultra-durable and permanent connections that are irreversible [23].

##### Ionic Hydrogels

The ionic groups attached to the polymer monomer formed polyelectrolytes hydrogels which are also known as ionic hydrogels [24]. Ionic hydrogels may be positive or negative or both positive and negative ends may be present in their polymer network. The charges might be positive or negative, defining the hydrogels. Either a positive or a negative charge combination. Sometimes cationic or anionic hydrogels results in an ampholytic macromolecule [25]. A significant swelling capability was observed in the anionic hydrogels with response to a change in the pH environment [26].

#### 1.1.5. Classification Based on Durability

Based on durability, hydrogels may be biodegradable or non-biodegradable. These categories are made based on their endurance. Natural hydrogels, such as agar, chitosan, and fibrin, are biodegradable, while Synthetic biodegradable polymers include polyanhydrides, poly(aldehyde guluronate), and poly(*N*-isopropyl acrylamide) [27]. Various vinylated monomers or macromeres, such as 2-hydroxyl ethyl methacrylate (HEMA), acrylamide (AAm), and 2-hydroxypropyl methacrylate (HPMA), are commonly used in the production of non-biodegradable hydrogels [28].

#### 1.1.6. Classification Based on Response to Stimuli

Many external factors affect the swelling and de-swelling properties of hydrogels. They go through a volume collapse or phase shift in response to various physical or chemical stimuli [29]. Based on the response to stimuli, hydrogels may be temperature sensitive, magnetic and electric field sensitive, solvent composition sensitive, pH and sound sensitive, and molecular species sensitive hydrogels [10].

## 2. Methods of Hydrogels Synthesis

Depending on the desired structure and use, hydrogels can be generated by establishing cross-linking in various ways. Grafting polymerization, physical cross-linking, chemical cross-linking, and radiation cross-linking are among the different preparation procedures used.

### 2.1. Physical Cross-Linking

Self-assembling hydrogels such as physically cross-linked hydrogels are generated by the molecular interaction of hydrophobic groups, hydrogen bonding, and electrostatic attraction followed by non-self-assembling of macromolecules. Physical interactions among distinct polymer chains inhibit the dissolution of physically cross-linked hydrogels [30]. The application of stress or variation in the physical environment disturbs these interactions because the physically cross-linked hydrogels can revert to their polymer chain. The physically cross-linked hydrogels are the emerging materials due to their facile synthesis and lack of a cross-linker. These cross-linking agents have an impact on the reliability of the substances to be imprisoned (e.g., cells, proteins, etc.) [30]. Various physical interactions are discussed below for the synthesis of physically cross-linked hydrogels.

#### 2.1.1. Ionic-Interaction

This approach is used to cross-link ionic polymers. For instance, the insertion of divalent or trivalent counter-ions can cross-link ionic polymers [31]. This approach is based on the idea of gelling a polyelectrolyte solution (for example, Na-alginate) with multi-valent ions of opposite charge (for example, Ca^2+^ + 2Cl^−^).

#### 2.1.2. Complex Coacervation

Complex coacervate gels can be produced by the interaction of a poly-anion with a poly-cation polymer. In this method, the opposite polymer charges cling together, forming soluble and insoluble complexes. These complexes are based on the solution pH and their concentration [32]. Coacervating polycationic chitosan with polyanionic xanthan is one such example. Positively charged proteins below their isoelectric point are more likely to bind with anionic hydrocolloids which result in the formation of polyion complex hydrogels [33].

#### 2.1.3. Hydrogen bonding

The degree of polymer functionalities, solution concentrations, temperature, and solvent type can be used to control the synthesis of hydrogels. Because of increased entanglements and hydrogen (H-)bonding interactions, hydrogels become more structured and stable by increasing the polymer concentration [34]. However, the network H-bonding becomes weak and disturbed within a few hours (h) by diluting the solution. For instance, a hydrogen bond is created between the –OH of PVA and the –NH_2_ group of adipic acid dihydrazide during the creation of the PVA and adipic acid dihydrazide hydrogel [35].

Hydrogels can be prepared by repeating the freezing and thawing processes, i.e., they are frozen and then melted at room temperature for several cycles. This mechanism includes the production of structures with microcrystals [36]. However, this method also relies on H-bond formation between the polymeric chains, and the most representative and widely studied polymer that can be freeze–thawed into a hydrogel is PVA [35].

#### 2.1.4. Maturation (Heat-Induced Aggregation)

In this method, the hydrogel is prepared by providing heat that induces the aggregation of the polymers. Gum Arabic (acacia gums) is mostly composed of carbohydrates, but it also contains 2–3% protein as an intrinsic element in its structure. The glycoprotein (GP) and arabinogalactan protein (AGP) make up gum Arabic. Heat-induced proteinaceous component aggregation raises the molecular weight, which results in the better mechanical characteristics and water holding capabilities of the synthesized hydrogels [37].

#### 2.1.5. Hydrophobic Interactions

An amphiphilic copolymers can be used to make the hydrogels by using an equilibrium part of hydrophilic and hydrophobic polymers [38]. Many hydrogels can be formed by the polymers cross-linking method via the hydrophobic interactions. When the polymer is heated and dehydrated, the interactions of hydrophobic units occurred with each other result in the formation of hydrophobic linkages. This linkage is facile because the heating process of the polymer solution accelerates the gelation process. These hydrophobic associations are supposed to cross-link the hydrogels. Diblock, triblock, and multiblock copolymers are examples of amphiphilic block copolymers. The nature of the polymer and the solution concentration of the polymer greatly influenced the characteristics of the amphiphilic block copolymer, because hydrogel is formed at an optimum concentration of the polymer solution [39,40].

### 2.2. Chemical Cross-Linking

To create permanent cross-linking in the hydrogels, the chemical cross-linking approach is preferred, which employs covalent interaction between polymer chains. Chemical cross-linking is the use of cross-linking agents or monomer grafting on the polymer backbone. Functional groups such as amino, carboxyl, and alcoholic groups containing synthetic and natural polymers that can be cross-linked by using the interaction between polymer functional groups and cross-linkers such as aldehyde groups. (e.g., glutaraldehyde, adipic acid dihydrazide, etc.). Enzyme catalyzed processes, polymer-polymer conjugation, and photosensitive agents accomplished the production of cross-linking [41]. Chemical cross-linking is carried out by the following methods.

#### 2.2.1. Schiff Base Reactions: Cross-Linking between Amino- and Aldehyde Groups

Cross-linking is a polymer chemistry stabilizing technique that results in multidimensional expansion of the polymeric chain, and ultimately, a network structure is resulted [42]. A cross-linker is a connection that connects two chains of polymers and may be covalent or ionic. Cross-linkers are made of two reactive functional groups and form a bridge between two polymeric chains. This approach entails the insertion of an additional molecule among the polymeric chains to develop cross-linked chains [43]. The most basic kind of cross-linking occurs between amino groups and aldehyde groups to generate a Schiff base. For example, the Schiff base reaction of dialdehydes such as glyoxal and, in particular, glutaraldehyde creates a covalent imine bond with the amino groups of chitosan. Because of its biocompatibility, genipin (a naturally produced compound from the fruit of gardenia) is commonly employed as a cross-linking agent alternative to dialdehydes. It has also been shown that genipin is covalently bound to polymers such as gelatin and chitosan [44]. A hydrogel made by cross-linking cornstarch and polyvinyl alcohol using glutaraldehyde as a cross-linker. The produced hydrogel membrane might be employed as artificial skin, and also deliver nutrients, healing elements, and medications to the targeted site [45].

#### 2.2.2. Polymer-Polymer Cross-Linking or Hybrid Polymer Networks (HPN)

The cross-linking process occurred in the hydrogels between the structural units of two polymeric chains that are linked to each other. As a result, reactive functional groups must be pre-functionalized in the polymers host. Depending on the rate of cross-linker, nature of functional groups, and biodegradability of the resultant product, led to the formation of various covalent connections among the polymer chain. This method allows the development of different types of linkages in the hydrogels network, relative biological inertness, and fast synthesis of diverse hydrogels [46].

#### 2.2.3. Photo Cross-Linking and Free Radical Polymerization

The presence of photosensitive functional groups is required for the formation of photo cross-linked hydrogels. When a photosensitive functional group is linked to a polymer, it can produce cross-links when exposed to UV light. Chitosan has received more attention than any other polymers among the photo cross-linking polymers [47]. By adding azide groups (–N_3_) to the polymeric chain of chitosan, a photo cross-linked chitosan hydrogel was developed. After being exposed to UV light, the azide group is changed to a nitrene group (R–N:), which binds the amino groups of chitosan molecule, resulting in the production of hydrogel in situ in a short time. Between the polymers, a photo cross-linked hydrogel can also form. Thermo-sensitive chitosan-pluronic hydrogel is one such example, in which both polymers were functionalized with photosensitive acrylate groups (H_2_C=CHCOO) through UV radiation. Thiol-ene photo-polymerization was used to create GO/PEG composite hydrogels [48].

#### 2.2.4. Cross-Linking by Chemical Reactions of Complementary Groups

Cross-linking polymer chains can involve chemical interactions involving complementary functional groups between cross-linkers and polymers. “Click” chemistry (e.g., Diels–Alder cycloaddition, azide-alkyne cycloaddition, thiol-Michael addition, etc.), Schiff-base reaction, condensation reaction, and many others are some typical examples [49]. Specific functional groups must be introduced into the polymer chains for each type of reaction. Azide-alkyne cycloaddition is a “click” reaction that occurs between an internal alkyne or terminal alkyne group and an azide group and has been used to make hydrogels [50]. Piluso et al., for example, synthesized alkyne-functionalized hyaluronic acid (HA). Another sort of “click” chemistry used in the creation of hydrogels is thiol-Michael addition. A thiol-Michael addition takes place between a thiol group and a variety of functional groups such as alkenes or alkynes, epoxy, vinyl sulfone, and maleimide [51]. To add the alkene groups, Liu et al. treated dextran using glycidyl methacrylate (GMA). After combining GMA-modified dextran with dithiothreitol (DTT) cross-linker, hydrogels may be easily produced in situ via thiol-Michael addition between thiol (from DTT) and methyl acrylate (from GMA-dextran) [52]. The amount of cross-linker (DTT) utilized may fine-tune the hydrogel’s biocompatibility, and that type of hydrogel can be efficient for use in 3D cell culture and cell transport applications [53]. Another sort of “click” chemistry that happens between a dienophile group and a diene is the Diels–Alder cycloaddition reaction. Tetrazine-norbornene and furan-maleimide are two common diene-dienophile complexes. The most popular method for creating hydrogels using Diels–Alder “click” chemistry is to first create precursors of hydrogels by altering polymers with the necessary functional groups. Cross-linking in polymers is used to generate hydrogel after mixing the hydrogel precursor solutions. For example, Desai et al. produced norbornene and tetrazine functionalized alginate by EDC/NHS coupling [54].

Free radical polymerization of a polymerizable group of hydrophilic polymers can result in chemically cross-linked hydrogels. The most commonly used cross-linking process for making hydrogels is free radical polymerization, which has the benefits of high reactivity, high conversion, and gentle reaction conditions [10]. Natural, synthetic, and semi-synthetic hydrophilic polymers were used to create gels by this method. The steps of free radical polymerization are as follows: initiation, propagation, and termination. For the initiation of polymerization, starters or initiators must be introduced to a system comprising vinyl monomers and cross-linkers to produce free radicals. Ammonium persulfate (APS) and azobis(cyanovaleric acid) (ACVA) are examples of common initiators. The system viscosity increases due to the propagation and cross-linking of polymer chains, which finally leads to gelation by stopping the polymerization process. Atmospheric oxygen acts as a free radical scavenger; therefore, the system is frequently purged with nitrogen gas [55].

### 2.3. Enzymatic Cross-Linking

This is a novel method for forming in situ hydrogels that uses an enzyme-catalyzed cross-linking process among polymeric chains. Tyrosinase, phosphopantetheine transferase, lysyl oxidase, plasma amine oxidase, and phosphatases were among the enzymes used for the synthesis of hydrogels. TG is a thiol enzyme that catalyzes the creation of extremely resistant covalent connections between a g-carboxamide group of a protein or peptide-bound glutamine and a free amine group of a protein or peptide-bound lysine [56,57,58]. Yung et al. (2007) created a hydrogel using microbial TG (MTG) as a cross-linker, having good biocompatibility and thermal stability and having the ability to deliver encapsulated regeneration cells (HEK293) in a regulated way. The most widely employed peroxidase enzymes in the creation of hydrogels are HRP and soybean peroxidase. In the presence of H_2_O_2_, they accelerate the conjugation of phenol and aniline derivatives. The HRP quickly binds with H_2_O_2_ in this process, and the resulting complex can oxidize hydroxyphenyl groups found in compounds such as tyrosine and tyramine [59]. Kim et al. (2011) recently created two-step HRP-catalyzed injectable tyramine modified hyaluronic acid (HA-Tyr) hydrogels [60]. The HA-Tyr conjugate was created in the first stage by forming an amide connection between the amine groups of tyramine and carboxyl groups of HA, and the HA-Tyr hydrogels were created in the second step by a radical cross-linking reaction using HRP and H_2_O_2_ [61]. This process is catalyzed by tyrosinases and oxidative enzymes. Chen et al. (2002) created gelatin gel using two methods: (i) cooling the gelatin solution and (ii) tyrosinase-catalyzed hydrogel creation. It was shown that the tyrosinase-catalyzed method for hydrogel synthesis improved the mechanical qualities [62].

### 2.4. Grafting

In this technique, a monomer is placed covalently on another polymer host molecule or grafting is the polymerization of monomer on a premade backbone. In this method, the polymer chain is activated by using radiation of high intensity or chemical reagents. The formation of functional monomers on activated macro radicals results in the branching and, ultimately, cross-linking of the monomer with the macromolecules [37].

#### 2.4.1. Chemical Grafting

During the chemical grafting, a chemical reagent activates the macromolecular backbones. Starch grafting with acrylic acid using *N*-vinyl-2-pyrrolidone is an example of chemical grafting [63]. Such hydrogels exhibit good pH-dependent swelling behavior and are appropriate for use as a medication and nutrient delivery method in the small intestine [64].

#### 2.4.2. Radiation Grafting

Electron beams or gamma radiation having high energy can start the grafting. Grafting of carboxymethyl cellulose (CMC) with acrylic acid using electron beam radiation results in the production of CMC hydrogels as described by Said et al. (2004) [65]. The free-radical polymerization of acrylic acid on the backbone of CMC was initiated using an electron beam. The product of water radiolysis will also be useful in extracting protons from macromolecular backbones. Irradiating both (CMC and monomer) generate free radicals that can combine to form a hydrogel [66].

### 2.5. Radiation Cross-Linking

Cross-linking by radiation is a popular technology since it does not require the use of chemical additives, and thus retains the biocompatibility of a polymer. Furthermore, the alteration and sterilization may be accomplished in a single step which makes it a cost-effective technique for modifying biopolymers with a specified end-use in biomedical applications [67]. The process is based primarily on the production of free radicals in the polymer because of their exposure to a powerful source of energy such as an electron beam, a gamma-ray, and an X-ray. The polymer environment (i.e., solid-state, dilute solution, and concentrated solution) will affect the radiation action (direct or indirect) [68].

## 3. Properties of Hydrogels

Because hydrogels are biodegradable, biocompatible, and non-toxic in situ, they have attracted a lot of interest in pharmaceutical and biomedical engineering. Some of their distinctive qualities, such as swelling behavior, mechanical properties, and toxicity tests must be evaluated before their use sector [69]. Some of the hydrogels’ qualities are as follows:

### 3.1. Swelling Properties

Hydrogels are internetworking materials that can expand in water and hold a large amount of water inside their structure without disintegrating. Swelling is the characteristic property of hydrogels, which depends on different environmental circumstances such as ionic strength, pH, and temperature [29].

**Figure 3:**
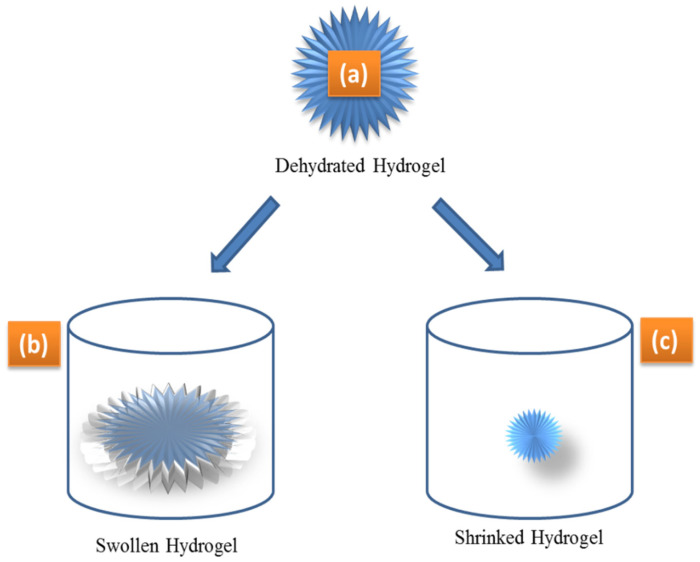
Dehydrated (**a**), swollen (**b**), and shrunken (**c**) hydrogels as a result of small changes in external stimuli, such as pH, temperature, and analyte concentration that influence the hydrogel hydrophilicity.

Hydrogels are made up of polymers that are cross-linked to one another through different cross-linking techniques. Therefore, they are all regarded as single molecules [70]. A little alteration in the environmental conditions can cause rapid and reversible alterations in hydrogel [71]. A variety of parameters, including cross-linking ratio, ionic environments, synthesis procedure, and polymeric chemical nature can influence the equilibrium and swelling kinetics. The swelling ratio, which is the weight-swelling ratio of swollen gel to dry gel is used to quantify the swelling characteristics of hydrogels. The swelling ratio of a hydrogel is affected by cross-linking with strongly cross-linked materials having a lower swelling ratio and vice versa [72]. Owing to the presence of hydrophobic and hydrophilic groups, the chemical structure thus affects the swelling behavior of hydrogels. Hydrogels that contain more hydrophilic groups swell more as compared to hydrogels that have hydrophobic groups. Temperature and pH also affect the hydrogels’ expansion. pH-sensitive hydrogels expand owing to the ionization of hydrophilic groups, which change with a change in the pH [73]. Electrostatic repulsion arises in the polymers chain, resulting in the breakdown of the polymer chain’s secondary bonding. The first phase in the expansion of hydrogels is water diffusion into the hydrogel network with the subsequent advance is the unwinding of polymer chains, while the development of hydrogel network is the third stage of hydrogel swelling characteristics. In a dried condition, the hydrogel is smooth, while in an expanded state, it is a rubbery material. When enough water enters the hydrogel framework, the shiny state changes into a rubbery condition known as expanding. The dissemination interaction is responsible for the passage and exit of water from the hydrogel matrix [74,75,76]. The swelling and de-swelling of hydrogels are shown in Figure 3.

### 3.2. Mechanical Properties

The mechanical properties of hydrogels involve their behavior under stress. The mechanical properties of hydrogels include tensile strength, percent elongation to break, toughness, and Young modulus. By varying the cross-linking degree, the desired mechanical characteristics of the hydrogel may be obtained. A stronger hydrogel might be created by increasing the degree of cross-linking. The higher the degree of cross-linking, the lower the percent elongation of the hydrogels, resulting in a more brittle hydrogel structure. As a result, there is an optimal degree of cross-linking to generate a moderately robust yet elastic hydrogel [77]. The mechanical characteristics of hydrogels are critical in pharmacological and biological applications. Mechanical property assessment is critical in a variety of biomedical applications, such as drug delivery matrix, ligament, tendon healing, and replacement material for cartilage. Hydrogels’ mechanical qualities should be such that they can retain their physical texture, wound dressing material, and tissue engineering while delivering therapeutic moieties for a specific period [78]. Compression and tension analysis, which can be accomplished by limited or unconfined local indentation with a probe or through frequency-based testing utilizing rheometry. There are two ways to determine the mechanical analysis of hydrogels. A rheometer is often used for frequency-based sinusoidal testing. The mechanical characteristics of calcium alginate hydrogel were evaluated by Grassi et al. [4]. The relaxation tests (at steady deformation, typical stress relaxation) were used to identify the linear viscoelastic range of the hydrogel and to describe the Youngs modulus and relaxation spectra using the extended Maxwell model. The density of hydrogels cross-linking might be calculated using the Youngs modulus and Flory’s hypothesis. This figure was then used to calculate the average polymeric mesh size based on the analogous network theory. As compared to the other traditional engineered materials, the investigation and characterization of the mechanical characteristics of hydrogels are extremely difficult because of differences in their mechanical properties. Hydrogels are appealing in biomedical applications due to their vast variation in mechanical properties. Hydrogels have a unique property known as poroelasticity. It is a time-dependent deformation that is independent of fluid flow. As well as this, hydrogels have two main mechanical properties, i.e., viscoelasticity and rubber elasticity. The elasticity in hydrogels is originated from the polymer matrix, while the aqueous phase-like water mobility in the network cage defines its viscosity. Applied mechanical force greatly affects the viscoelastic properties. We can say that the viscoelastic properties of hydrogels are dependent on the applied stress. The stress is proportional to the strain in the elastic material, while on the other hand, the stress is proportional to the strain rate for viscous materials. For linear viscoelastic properties, a mathematical model is developed known as the Maxwell model [12].

The dynamic mechanical analysis was also performed to investigate the viscoelastic properties. In this method, a sinusoidal load is applied in shear mode on one of the sides of the hydrogels. The strain transducer is used for measuring the change in length of the sample while the stress is calculated with the stress transducer [79]. The sinusoidal response is described in Equation (1).

G = G′ + iG″(1)

G″ is the imaginary modulus, while the G′ is the real elastic modulus.

Based on their hydrophobic properties, hydrogels’ mechanical properties can be altered by verities of techniques. Grafting or surface coating on stronger support can also improve and modify the elasticity and strength of hydrogels. Another method is the introduction of other materials, which results in the formation of super-porous hydrogels composites. Nanoparticles’ introduction can also modify their gelation and other properties which make them suitable for their use in the biomedical field [80].

### 3.3. Biocompatible Properties

Biocompatibility refers to a material’s ability to be used in a certain application without causing a host reaction [81]. Biocompatibility is made up of two components, i.e., bio functionality and bio-safety. Bio-safety is the right host response that is not only systemic, but also local, the lack of cytotoxicity, mutagenesis, and/or carcinogenesis, and bio-functionality is the capacity of a substance to do the precise task for which it is designed [35]. This term is especially important in tissue engineering since tissue constructs constantly interact with the body throughout the healing and cellular regeneration processes, as well as during scaffold breakdown. If this need is not satisfied, the hydrogel may get fouled, or there may be injury and scarring to related issues, whether they are directly adjacent or linked by the vasculature. In vitro cell, culture techniques, which are commonly used to check the tissue compatibility of implanted devices can be used to assess biocompatibility. Cell culture procedures are frequently referred to as cytotoxicity testing. To assess the biocompatibility of the hydrogels, three major cell culture tests are used: elution (extract dilution), direct contact, and agar diffusion. Hydrogels must be biocompatible and nontoxic [82]. Many synthetic polymers are non-toxic and biocompatible with live cells. On HeLa cells, the cytotoxicity of poly (prop acrylic acid), poly glutamic acid, poly (methacrylic acid), poly (acrylic acid), and poly (methacrylic acid) hydrogels were tested, and these hydrogels demonstrated 90% cell survival [83]. The majority of polysaccharides are found to be biocompatible and useful as biomaterials. Furthermore, while synthetic hydrogels are non-toxic to some extent, they have lower cytocompatibility than natural polysaccharides [83].

### 3.4. Biodegradability

The use of hydrogels having biodegradability is critical in the biomedical area. The degradation of hydrogels into harmless byproducts by living organisms is referred to as biodegradability [84]. The biodegradability of hydrogels is determined by the moieties in the systems as well as the manner of preparation. Solubilization and hydrolysis of biological entities of hydrogels into final products are examples of degradation processes [85]. The hydrogels may degrade and be cleared from the body by bio-erosion and bio-absorption. Hydrophilic, natural, and synthetic polymers are all examples of biodegradable polymers. Water absorption caused substantial swelling of the hydrogels, which eventually dissolved due to insufficient water absorption [86]. The degradation of these polymers is influenced by several parameters, including hydrophilicity and polymer–water interaction [87]. Other elements, such as temperature and pH, might affect hydrogel breakdown through simple solubilization. Chemical hydrolysis can destroy some polymers that cannot be biodegraded by simple hydrolysis. Some polymers cannot be biodegraded by simple hydrolysis but can be destroyed via chemical hydrolysis [88]. Furthermore, hydrogels can be destroyed by enzyme hydrolysis, which includes polymers such as polysaccharides, proteins, and synthetic polypeptides [89]. These polymers can be exploited in drug delivery systems due to the great benefit of enzymatic breakdown. Enzyme hydrolysis takes place via the hydrolysis groups, which catalysis the hydrolysis of C-N, C-C, and C-O bonds. Proteinases and peptidases are hydrolases enzymes that dissolve the polypeptide and protein hydrogels, respectively [90]. Chitosan can be destroyed through glucosamine-glucosamine, glucosamine-*N*-acetyl-glucosamine, and *N*-acetyl-glucosamine-*N*-acetyl-glucosamine enzyme hydrolysis. However, chitosan breakdown also happens via free radical and redox processes [91,92,93].

## 4. Characterization of Hydrogels

Hydrogels are distinguished by their shape, swelling property, chemical structure, and elasticity. The existence of hydrogels in a system may be easily quantified by dispersing the polymer in water in a cylindrical vial and visually seeing the development of insoluble material. Turning the universal upside down to observe the solution viscosity can also offer a rapid assessment of the bulk viscosity. The following are the key characteristics for identifying hydrogels.

### 4.1. Solubility

#### 4.1.1. Method A

Typically, the hydrogel content of a specific material is evaluated by calculating and measuring the insoluble component of the dried sample by immersing it for 16 or 48 h at room temperature in deionized water. To ensure the uniform distribution of hydrogel material in water, the sample should be produced at a dilute concentration (usually 1 percent) [94]. The gel fraction is calculated as follows (Equation (2))

(2) $$(\mathrm{Gel}\;\mathrm{Fraction}\;\mathrm{Hydrogel}\;\%)=\frac{W_{d}}{W_{i}}\times 100$$

where W_i_ denotes the original weight of the dried sample, and W_d_ denotes the weight of the dried insoluble fraction of the sample after water extraction.

#### 4.1.2. Method B

The weight maintained after vacuum filtering can be used to get a more precise measurement of the insoluble fraction, commonly known as hydrogels. The JECFA (Joint Expert Committee on Food Additives) method for hydrocolloids is based on method B, except with the solvent changed from mild alkaline to water. After drying at 105 °C for 1 h in an oven and cooling in desiccators possessing silica gel, the weight (W_1_) fiberglass of 70 mm fiber paper (pore size 1.2 micron) is calculated. Depending on the test material, a 1–2 weight percent (S) dispersion in distilled water can be made, followed by hydration overnight at room temperature. Before filtering for 2–5 min, the hydrated dispersion is centrifuged at 2500 rpm. The filter paper is dried in an oven at 105 °C before being cooled to a consistent weight (W_2_) [37]. The percentage insoluble can then be calculated by Equation (3).

(3) $$\%\;\mathrm{Insoluble}\;\mathrm{Hydrogel}=\frac{W_{2}-W_{1}}{S}\times 100\;$$

Depending on the type of test material, a variable mesh size, such as a 20-mesh steel screen (1041 m), can be employed for gel fraction determination [95].

### 4.2. Swelling Measurement

Swelling in hydrogels may be measured using three distinct approaches. The Japanese Industrial Standard K8150 technique was utilized to quantify the swelling (expansion) of hydrogels for this purpose. The dried hydrogels are submerged at room temperature for 48 h in deionized water on a roller mixer. After swelling, the hydrogels are filtered through a stainless-steel net with a 30-mesh size (681 m). To evaluate the swelling of hydrogels in a second approach, dried hydrogels (0.05–0.1 g) are distributed in a sufficient enough amount of water (25–30 mL) for 48 h at 25 °C in a volumetric vial (Universal). After that, the mixture is centrifuged to separate the water-bound material layers from the unabsorbed water. The free water is eliminated, and the swelling is quantifiable [94] and calculated by Equation (4).

(4) $$\mathrm{Swelling}=\frac{W_{s}-W_{d}}{W_{d}}$$

where W_s_ and W_d_ are the weight of hydrogels in the swollen and dry state, respectively. For more or less equivalent measures, the terms’ swelling ratio [94], equilibrium degree of swelling [96], or degree of swelling [97] have been employed.

Another way for measuring swelling is to use the Japanese Industrial Standard (JIS) K7223. At room temperature, the dried gel is submerged for 16 h in distilled water. After swelling, the hydrogels are filtered through a stainless-steel net with a 100-mesh size (149 m) [37]. The following formula is used to determine swelling (Equation (5)) [98].

(5) $$\mathrm{Swelling}=\frac{C}{B}\times 100$$

where C and B is the weight of the hydrogels produced after drying and insoluble component acquired after water extraction.

### 4.3. FTIR

FTIR (Fourier Transform Infrared Spectroscopy) is a valuable method for determining a substance’s chemical structure. It deals with the fundamental constituents of a material, i.e., chemical bonds can generally be activated and absorb infrared light at frequencies characteristic of the chemical bond types [99]. The resultant IR absorption spectrum is a fingerprint of the substance being analyzed. This approach is commonly used to compare the structural sequence of hydrogels to the starting materials [100].

### 4.4. Scanning Electron Microscopy (SEM)

SEM may be used to determine the surface topography, composition, and other attributes of a sample, such as electrical conductivity [101]. Magnification in SEM may be adjusted over a six-order-of-magnitude range, from around 10 to 500,000 times [102]. This is an effective approach for capturing the distinctive ‘network’ structure of hydrogels [103,104].

### 4.5. Rheology

The rheological characteristics of a system are heavily influenced by the kinds of structure (i.e., association, cross-links, and entanglement) present. At low frequencies, solutions of polymer are fundamentally viscous, leading to obey the scaling laws: G′ and G″. At high frequencies, elasticity takes precedence (G′ > G″) [105]. This is related to the single-relaxation-period of Maxwell-type behavior that can be calculated from the crossover point and that grows with concentration. G′ and G″ for cross-linked microgel dispersions are nearly independent of oscillation frequency. This approach has been used to analyze the structure of the network of seroglucan/borax hydrogels, cationic hydrogels based on chitosan, and a variety of other hydrocolloids [105,106,107]. Varieties of rheological tests have been reported in the literature. The strain sweep test is one of them, which is used for the characterization of hydrogels by keeping constant frequency and rising oscillatory strain. The storage (G′) and loss (G″) moduli of the hydrogel across an increasing strain range are used to represent the outcome of a strain sweep test, which also provides information on the object’s Newtonian characteristics or linear viscoelastic region (LVR). The primary goal of strain sweep testing is to identify the hydrogels’ linear viscoelastic region. Similarly, the time sweep test is also a rheological characterization technique used for the investigation and alteration in the structure of hydrogels with respect to time. To study the gelation process and stability of hydrogels, the time sweep test is the right technique [108].

### 4.6. X-ray Scattering Techniques

X-ray scattering techniques are a group of analytical procedures that provide information about a material’s chemical composition, crystal structure, and physico-chemical properties. The scattered intensity of an X-ray beam striking a sample is measured as a function of energy, wavelength, scattered angle, and polarization. X-ray diffraction is now often considered a sub-set of X-ray scattering, where the scattering is elastic and the scattering object is crystalline, so that the resulting pattern contains sharp spots analyzed by X-ray crystallography. However, both scattering and diffraction are interrelated and the distinction has not always existed. Diffraction analysis is used to determine the crystallinity of a substance, i.e., if the material is amorphous or crystalline. It is used to determine whether the polymers keep their crystallinity structure during the processing pressurization procedure. Diffraction analysis is a prominent method for determining the morphology of hydrogels [109,110,111]. The other types of scattering techniques for hydrogel characterization includes X-ray fluorescence and small/wide angle X-ray scattering. For advanced hydrogel characterization, small-angle neutron scattering (SANS) and dynamic light scattering are used. These advanced experimental methods examines the structure of hydrogels at a mesoscopic scale of different range by using various sub-atomic particles at either small or wide scattering angles [109,110,111].

### 4.7. SANS

SANS is an experimental method that examines the structure of diverse substances at a mesoscopic scale of around 1–100 nm by using elastic neutron scattering at small scattering angles. To investigate the microscopic and nano structure of the hydrogels, the X-ray and neutron scattering techniques were used to explore the formation of a porous network in which polymer crystallites serve as connection sites during a freeze/thaw gelation process. T. Puspitasari et al. reported the SANAS analysis of polyvinyl alcohol (PVA). For this purpose, the crystallites were considered to be polydisperse and homogeneous spheres fitted using a theoretical spherical model computation in order to analyze the crystalline phase. They discovered that the number of freeze/thaw cycles had a significant effect on the size and dispersion of crystallites. The size of crystallites was decreased by irradiation at the same number of freeze/thaw cycles, with similar results [112].

## 5. In Vitro Drug Release Study

Because hydrogels are inflated polymeric networks, the interior might be filled by various drugs molecules, and targeted delivery studies are conducted to understand the process of release concerning time. The parameters are compared with the standard plot to determine the equivalence of the medication solutions [9].

### Spreadability Study

The spreadability test is performed using an instrument comprised of two glass slides with a pan fixed on a pulley and a wooden block with a scale. Among two wood glass slides, the excess formulation is sandwiched and a 100 g weight is put on the upper glass slide for the comparison of the formulation’s thickness. Weight can be added, and the time it takes to separate the two slides is used to calculate spreadability (Equation (6)).

(6) $$S=\frac{m\;\times \;l}{t}$$

where S is the spreadability, m is the weight linked to the top slide, l is the length of the glass slide, and t is the time required in seconds [113].

## 6. Biomedical Applications of Hydrogels

**Figure 4:**
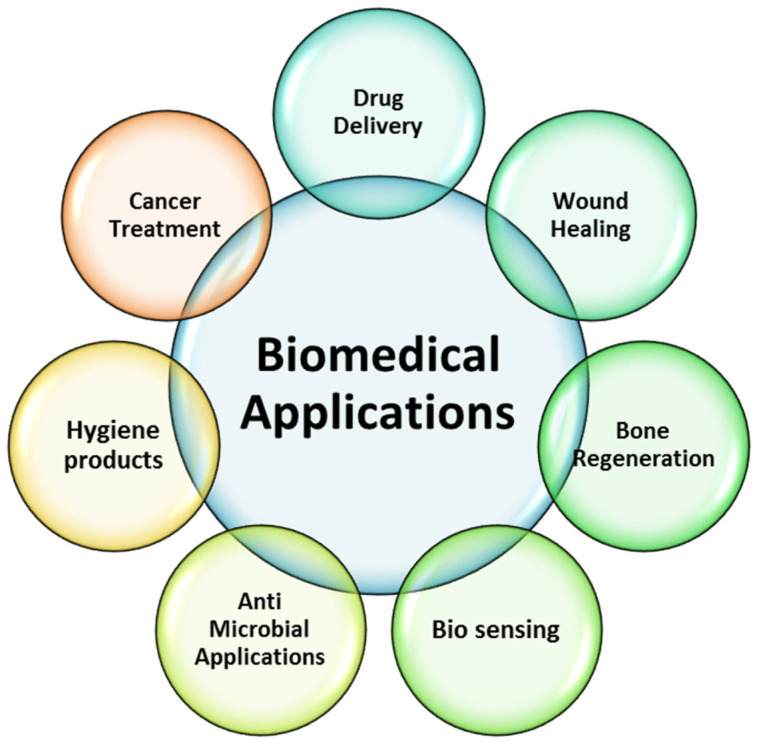
Biomedical applications of hydrogels.

Hydrogels have a wide range of applications, due to their distinct designs and ability to work in different environments. Because of their water content, hydrogels are flexible enough to be employed in a wide range of industrial and biological applications. Some of the biomedical applications of hydrogels are shown in Figure 4.

### 6.1. Hydrogel for Drug Delivery

Controlled drug delivery systems (DDS), which distribute medications at predetermined rates for certain periods have been employed to circumvent the constraints of traditional drug formulations [114]. The affinity of hydrogels to the aqueous environment in which swelling occurs and the degree of cross-linking in the matrix can result in high porosity hydrogel structures. Because of their porous architecture, hydrogels are extremely permeable to many types of medicines, allowing medications to be delivered to the specified under controlled conditions [10]. In drug delivery research, hydrogels’ ability to release (sustained-release) medicinal goods over a long period is a significant benefit, resulting in high concentrations of active medicinal material in specified locations over extended periods. To increase the binding between the loaded drug and the hydrogel matrix and extend the time of drug release, both physical (electrostatic interaction) and chemical (covalent) approaches can be applied [88]. Hydrogels can store and preserve certain medications from harsh conditions while also releasing them at the right kinetics. Local variations in pH, temperature, the presence of certain enzymes, or distant physical stimuli may cause drug release on demand. Porous covalently cross-linked permanent network hydrogels have pores and these holes contain water molecules that are either free or attached to the hydrophilic groups of networks, and they are employed for drug loading and unloading. This drug loading and unloading are functioned by a change in stimulus. Ionically cross-linked hydrogels administer drugs by alerting the pH [88]. The loading and unloading of drugs result in the swelling and shrinking of hydrogels. The swelling of hydrogels diminishes as the cross-linking density increases, resulting in the stability of the polymer network. The increased cross-linking density of the polymer network leads to a reduction in drug release [115]. Swelling-controlled release of drugs using hydrogels use medicines placed inside a glass polymer that starts swelling when in touch with a bio-fluid. Swelling caused the polymer chain to relax and expand beyond its border, allowing the medication to diffuse. The method, also known as Case II transport, allows for time-independent, continuous drug release kinetics. Because the slope between both the distributed therapeutic agent in the hydrogel and its local environment allows active pharmaceutical ingredient diffusion from a higher concentration region inside the hydrogel to a lower concentration region, the process is commonly called as unusual transport because it integrates both the diffusion and swelling processes to allow release of the drug. Diffusion-controlled delivery of drugs with hydrogels employs reservoir or matrix technology to allow drug release by diffusion via a hydrogel mesh or holes water filled. The hydrogel membrane is coated over a drug-containing core in the reservoir delivery system, generating capsules, spheres, or slabs with a high drug concentration in the very center of the system to promote a consistent drug-release rate. The matrix method operates through macromolecular holes or mesh, whereas the reservoir delivery system delivers time-independent and consistent drug release. Bio-inspired hydrogels are a newer type of hydrogel utilized for medication delivery applications. These 3D materials recreate the biological microenvironment relevant to the medical state and aid in research on how to improve the targeted drug delivery mechanism, how the therapy performed in vivo, how the illness proceeded, and so on. These are especially effective in cancer therapy since the disease is notoriously complicated, with numerous molecular and physiological alterations that necessitate ongoing monitoring.

### 6.2. Tissue Engineering (TE)

In vivo tissue regeneration is particularly significant. The patient’s cells are blended with the polymer in vitro until they are ready to be implanted [116]. The hydrogels function as a natural extracellular matrix, promoting cell proliferation and tissue regrowth. The pseudo-extracellular matrix, which is made of growth factors, metabolites, and other components, connects cells and governs tissue structure, with the ultimate objective of replacing missing or injured natural tissue. Numerous therapeutic options are available when tissues or organs fail in portions, including repair, replacement with a synthetic or natural alternative, and regeneration [116,117,118]. Tissue repair or replacement with a synthetic alternative is only possible in cases when surgical procedures and implants have proven effective. Although implants have been somewhat effective, tissue engineering offers enormous capacity for the regeneration of failing tissue. Complete replacement of the sick or dysfunctional organ or tissue with a natural substitute necessitates transplantation of an appropriate and healthy alternative. Thus, tissue engineering has considerable potential for organ regeneration [119].

### 6.3. Bone Regeneration

Bone self-recuperating is restricted and generally needed outer intercession to enhance bone fix and recovery [120]. While ordinary methodologies for recuperating bone anomalies such as autografts, allografts, and xenografts have been generally utilized. They all have disadvantages that confine their functional use. Despite the advancement of a wide scope of biomaterials, for example, metal inserts, calcium phosphate concretes (CPC), hydroxyapatite, etc., the expected remedial effect is not figured out [120]. Hydrogels are those polymeric platforms that are increasingly attracting attention, and their unusual arrangements and flexible physicochemical properties have been thoroughly explored. Hydrogel-based cell circulation and drug organization have emerged as viable options for tissue design and regenerative medicine to improve bone repair work. They can create a hydrophilic three-dimensional environment that promotes cell endurance. Furthermore, hydrogels can be tailored to offer the desired shape for implantation or infusion, and the rate of debasement, porosity or delivery profile can be quickly controlled by adjusting the degree of cross-linking and synthesis techniques [120,121,122].

### 6.4. Applications in Spinal Cord Regeneration

Spinal line injury (SCI) is a troublesome recovery challenge. A significant number of these lesions do not hurt the dura mater, and a portion of the axons remain alive and can be recovered at the harm site. In such cases, surgically placing a prefabricated frame or DDS into the injured spinal cord may result in a future injury. The usage of in situ forming scaffolds is one option for this procedure. After injecting DDS into the affected cord region, viscoelastic hydrogels quickly convert from liquid to gel and conform to the tissue at the injury site [76]. In vivo conversion of fluid hydrogels to the gel, the structure will bridge the small split between spinal string tissue and, surprisingly, cut across segments created after SCI. The gel, which currently serves as a platform, will minimize voids and lay the groundwork for the wounded rope tissue’s recuperation by assisting cell ingress and framework. As a result, there is no compelling necessity to manufacture pre-assembled frameworks for each harm site separately, and alienating tissue at the injury location to embed the pre-arranged platform is avoided, which can cause greater harm and loss of function [123,124].

### 6.5. Biosensors

A biosensor is made by consolidating physical and synthetic sensors. A biosensor may be viewed in two ways: as a gadget that can detect and report a biophysical quality of the framework being scrutinized, or as a gadget that can offer pertinent insightful data from changing biochemical information [125]. The incorporation of a natural acknowledgment part, which considers the investigation of organic information, is a component shared by all biosensors. Biosensors are turning out to be more critical as useful instruments for a wide scope of utilizations, for example, the reason behind care testing, home diagnostics, and ecological observing. The organic acknowledgment area, known as the bio component, is comprised of many designs such as chemicals, antibodies, live cells, or tissues, yet the thought is that it is exceptional to one analyte and has no response to another meddling. Capture into layers, physical adsorption, ensnarement into a framework, and covalent holding are on the whole procedures for connecting bio-atoms with sensors [126,127]. Hydrogels are inherently biocompatible because of their high-water content and hydrophilic person, which are similar to the void-filling part of the extracellular framework. Subsequently, one clear utilization of hydrogels in biosensors is the insurance and covering of sensor components to keep away from undesirable contact with natural atoms or cells. Hydrogels might be utilized as bio detecting component immobilization lattices and give ideal conditions to proteins and other biomolecules to keep up with their dynamic and practical construction. The cooperation among analyte and sensing components causes a volume change in light of the objective part, and this volume change is the reason for ID in hydrogel-based sensors [128,129,130].

### 6.6. Applications in Wound Healing

Hydrogels are cross-linked polymers that can be natural or synthetic and are employed in a range of biological applications. They are comprised of a framework of insoluble polymers with a water content of approximately 96%. These hydrogels can contribute water to the wound site, assisting in the maintenance of a moist environment, resulting in quicker wound healing [131]. These are used to make drug-delivery vehicles, contact lenses, wound dressings, and electrodes or sensors. The wound healing process can be speed up by inducing some factors by applying many agents. Hydrogels are one of them. Hydrogels can induce the factors which accelerate the wound healing and tissue regeneration process [4]. Verities of therapies have been practiced till now for this purpose. However, the desired result was still not achieved. Therefore, modern therapies are required. The use of hydrogels can solve this issue. The necessary conditions which operate the healing process are the proper moister and barrier against micro-organisms. Hydrogels are found to be efficient regarding the stated factors. Hydrogels contain enough water which keep the wound moist. On the other hand, hydrogels show significant protection against microbial attacks. These two main factors make hydrogels efficient and advance alternative therapy for wound healing and wound dressing. Aquaform, Intrasite, GranuGel, Nu-Gel, Purilon, and Storage are examples of hydrogels. These hydrogels may also absorb a certain amount of wound exudate. They transport moisture, vapor, and oxygen, although their bacterial and fluid permeability is determined by the secondary dressing employed. The association, dissociation, and binding of different ions to polymer chains cause hydrogels to expand or shrink in aqueous solutions. These frameworks can expand in water until they arrive at a harmonious state and afterward return to their unique shape [132]. The collaborations liable for water sorption by hydrogels incorporate the course of hydration, which is associated with the presence of such compound gatherings as -OH, -COOH, -CONH_2_, -CONH-, and -SO_3_H and the presence of fine regions and contrasts in osmotic strain. The existence of covalent connections between individual polymer chains, as well as hydrophobic and electrostatic interactions, makes hydrogel dissolution difficult. In comparison to typical gauze treatment, the use of hydrogels appears to greatly enhance wound healing. Hydrogel wound dressings are produced using an assortment of normal and manufactured polymers that are biocompatible. Normal polymers, for example, alginate, chitosan, gelatin, and collagen are instances of these polymers, as are manufactured polymers such as polyurethane, poly (ethylene glycol), polycaprolactone, poly (vinyl pyrrolidone), poly(lactide-co-glycolide), polyacrylonitrile, poly(amino corrosive), and others [133].

### 6.7. Hygiene Products

Superabsorbent hydrogels, particularly those based on acrylates, are broadly used in cleanliness items to retain liquids. They can keep dampness away from the skin, advancing skin wellbeing, avoiding diaper rash, and making utilization more agreeable. Disposable diapers containing superabsorbent polymers (SAPs) are used by parents in all developed nations as well as hospitals worldwide. The usage of these materials is expected to rise further in the training pant and adult incontinence product industries. SAPs can help limit germ colonization, lowering the danger of fecal contamination and the spread of gastrointestinal illnesses [134]. In 1982, Caló E suggested the first application of SAPs in the diaper sector, with its following usage in sanitary napkins [135]. Diapers became slenderer and better for water maintenance. It became achievable to fabricate diapers with a maximum spillage of under 2% and to bring down the typical load of a medium-size diaper by around half, with clear advantages as far as environmental concerns and diminished creation costs. Making recyclable dispensable diapers, napkins, clinic bed sheets, clean towels, and other comparable products is subsequently a basic objective for the present-day business. An original answer for this issue has recently been proposed, which incorporates the utilization of biodegradable cellulose-based hydrogels. Hydrogels consolidating sodium carboxymethylcellulose (CMC) and hydroxyethyl cellulose (HEC) cross-connected with divinyl sulfone (DVS) may enlarge like SAPs and have huge water maintenance under outward burdens [135]. These headways were made conceivable by including miniature permeable designs into the hydrogels, which lift water maintenance and expand energy attributable to capillarity impacts [135]. One of the main hydrogel-framing polymer creations appropriate for the production of cleanliness products is portrayed in US Patent 32,649, which talks about one of the principal hydrogel-shaping polymer structures. It is comprised of a water-insoluble, to some degree cross-connected polymeric substance that might be produced using carboxylic acids and corrosive anhydrides or olefinically unsaturated sulfonic acids by free-extremist polymerization in a watery arrangement within the sight of a cross-linking agent [135].

When exposed to water or organic liquids, this material can be dried to produce polymer structures that can form hydrogels. Osborn introduced a female sanitary napkin in US Patent 5,009,653. The center was created utilizing acidic monomers such as acrylic corrosive, methacrylic corrosive, or 2-acrylamide-2-methyl propane sulfonic corrosive to create a hydrogel-shaping substance. This material was extremely permeable, and resists the menstrual flows, and was remarkably comparable to the body, reducing the possibility of spills and stains [135].

### 6.8. Applications in the Treatment of Cancer

Depending on the cancer patient’s diagnosis and stage, several approaches and procedures are performed. Treatment options include chemotherapy, immunotherapy, radiation therapy, surgery, targeted therapy, and gene therapy. Chemotherapy is a major component of these techniques for tumor control and recurrence prevention. Chemotherapeutic drugs’ anti-tumor activity involves drug toxicity, which kills cancerous cells. If targeted or focused killing is not performed, chemical treatments may damage normal tissues as well as cancer cells [136].

The development of hydrogels has given traditional chemotherapy treatments a new lease on life. Drugs are delivered directly into or around the tumor through the hydrogels. Drugs could be contained within a cross-linked 3D network of hydrophilic polymer chains, limiting toxicity within a specific location where tumor cells reside. Meanwhile, localized hydrogels have the ability to deliver medications to the tumor site in a constant and efficient manner [137]. To improve this property, a variety of systems with varied compositions, such as polyphosphazene (PPZ), polyethylene glycol (PEG), and polylactate glycolic acid, (PLGA) have been developed [138].

Traditional intravenous chemotherapy involves a variety of systemic side effects, including myelosuppression, hepatic or renal failure, and neurotoxicity. As an alternative, injectable hydrogels can efficiently avoid these difficulties by administering drugs topically to the tumor site. Because of the benefits of localized drug toxicity in the tumor site, proper injectable hydrogels as a drug delivery mechanism has become a research focus [139]. Based on different types and stages of cancer, a variety of hydrogel drug delivery systems have been developed, including photosensitive, thermosensitive, pH-sensitive, and dual-sensitive hydrogels [139].

### 6.9. Anti-Fungal Applications

Academics are interested in hydrogels containing antifungal drugs because of their potential use in several applications, such as wound dressing and ultrasound pads [137]. Fungi are quickly gaining a reputation as significant pathogens in bloodstream infections, which usually include in-dwelling devices. Antifungal properties in materials could be a substantial deterrent to these infections [140]. Various hydrogels have been developed that have high antifungal properties and may be utilized against a variety of fungi. For example, Andreas Zumbuehl et al. developed dextran-based hydrogels that are efficient against Candida albicans [141]. Abousamra et al. developed nystatin-based hydrogels for the treatment of topical candidiasis [142].

### 6.10. Anti-Bacterial Applications of Hydrogels

Hydrogels have been studied extensively as a potential antibacterial agent. Hydrogels with the appropriate properties, such as porosity and hydrophilicity, can be made for antibacterial applications by carefully selecting monomers and cross-linkers. In addition, some hydrogels have an inherent antibacterial property [143].

Based on the classification of hydrogel matrices and antibacterial agents, antibacterial hydrogels are divided into three categories: (i) hydrogels containing inorganic nanoparticles, (ii) antibacterial agents, and (iii) hydrogels with intrinsic antibacterial properties [144]. Metallic oxide nanoparticles are the most common inorganic antibacterial compounds. Metals/metal ions that are commonly utilized include, yet, are not restricted to, silver (Ag), gold (Au), and copper (Cu). Zinc oxide (ZnO), titanium dioxide (TiO_2_), and nickel oxide are instances of metallic oxide metal nanoparticles that are utilized. Silver nanoparticles (Ag NPs) and zinc oxide nanoparticles (ZnO NPs) are now the most extensively employed inorganic antibacterial materials [145]. Inorganic antibacterial material stacked hydrogels not only improve antibacterial capabilities but also preserve antibacterial activity over time, reducing the possibility of bacterial resistance [143].

### 6.11. Hydrogels in Contact Lens

Hydrogels can be used in artificial contact lenses. The requirements which are necessary for an efficient contact lens are fulfilled by typical hydrogels. They keep the eye healthy and white because it easily allows oxygen to pass through. Polyvinyl alcohol (PVA) is a synthetic polymer having many alcoholic functional groups. It possesses considerable hydrophilic character and biocompatibility. PVA hydrogels can be a synthesized polymer solution in the presence of water as a solvent. PVA can be modified with cyclic acetal group followed by cross-linking with other PVA which results in the formation of cross-linked hydrogels. Pure PVA contact lenses without any modifications are found to be suitable for contact lenses. For modifications, PVA has been used as a tool/precursor for the formation of more efficient and comfortable contact lenses. Approximately 47,000 molecular weight PVA can lubricate the eyes because it can leach out of the lens. These properties made hydrogels suitable for corneal replacement and ophthalmic application. Hyaluronic acid (HA)-modified hydrogels are considered efficient for contact lens applications. This material is used widely for tissue engineering purposes. The hydroxyl and amino group present in each repeating unit of HA are responsible for their hydrophilic nature which makes it suitable and biocompatible for incorporating into a contact lens.

By employing a small amount of HA, one can minimize the water contact angle. HA-modified hydrogels can also limit lysozyme absorption. The use of high molecular weight HA causes a difficulty with transparency. This disadvantage, however, can be easily solved by employing HA with a low molecular weight. The HA covalently connected HA to HEMA lenses have a transparency of more than 92% and a low rate of contact lens dryness. HEMA lenses can be covalently changed with HA using UV radiation, resulting in a decrease in lysozyme absorption and water contact angle. Weeks et al. used UV light to covalently attach a methacrylate-modified HA to HEMA and silicone hydrogel lenses. In all cases, the HA-modified hydrogels had a considerably reduced water contact angle and lysozyme absorption. They also noted that HA with little or no methacrylation on the surface would not prevent the protein from connecting with the hydrogel’s surface, but that if HA was entrapped inside the hydrogel, the protein would have fewer opportunities to interface with the surface, preventing its deposition.

## 7. Conclusions

This review summarizes the introduction of hydrogels, their classification, structure, cross-linking, and modifications. Furthermore, a detailed classification based on different factors has also been discussed. The biomedical applications of hydrogels are also encountered, with special emphasis on controlling drug delivery. Hydrogels are a large network of small monomers cross-linked to each other physically or chemically. Biocompatibility and biodegradability are two of their most impressive features, making them an excellent alternative for biological and environmental applications. Hydrogel design has advanced substantially in recent decades, resulting in a wide range of applications in the pharmaceutical, biomedical, and food industries, among others. In pharmaceutical therapeutic administration, hydrogels are available in a range of dosage forms, including tablets, capsules, wound dressings, transdermal films, and so on. Different procedures, such as cross-linking, can be used to create them. They have a wide range of applications in every sector, particularly in biomedicine. Different nanomaterials can be included into hydrogels to make them suited for specific uses. They can be made to respond to a certain stimulus at a specific level, such as light, pH, electric field, temperature, and so on, making them stimuli sensitive.

## Data Availability

Not applicable.
